# The 2014 liver ultrasound tracking benchmark

**DOI:** 10.1088/0031-9155/60/14/5571

**Published:** 2015-07-02

**Authors:** V De Luca, T Benz, S Kondo, L König, D Lübke, S Rothlübbers, O Somphone, S Allaire, M A Lediju Bell, D Y F Chung, A Cifor, C Grozea, M Günther, J Jenne, T Kipshagen, M Kowarschik, N Navab, J Rühaak, J Schwaab, C Tanner

**Affiliations:** 1 Computer Vision Lab, ETH Zurich, 8092 Zurich, Switzerland; 2 Computer Aided Medical Procedures, Technische Universität München, 80333 München, Germany; 3 Konica Minolta Inc., Osaka 550-0005, Japan; 4 Fraunhofer MEVIS Project Group Image Registration, 23562 Lübeck, Germany; 5 Fraunhofer MEVIS, 28359 Bremen, Germany; 6 Medisys Lab, Philips Research, Suresnes, France; 7 Engineering Research Center for Computer-Integrated Surgical Systems and Technology, Johns Hopkins University, Baltimore, MD 21218, USA; 8 Institute of Biomedical Engineering, University of Oxford, UK; 9 Department of Radiology, Churchill Hospital, Oxford, UK; 10 Fraunhofer FOKUS, 10589 Berlin, Germany; 11 Angiography & Interventional X-Ray Systems, Siemens Healthcare, Forchheim, Germany; 12 Mediri GmbH, 69115 Heidelberg, Germany; vdeluca@vision.ee.ethz.ch

**Keywords:** respiratory motion, ultrasound, tracking, image registration, image guidance, motion estimation, challenge

## Abstract

The Challenge on Liver Ultrasound Tracking (CLUST) was held in conjunction with the MICCAI 2014 conference to enable direct comparison of tracking methods for this application. This paper reports the outcome of this challenge, including setup, methods, results and experiences. The database included 54 2D and 3D sequences of the liver of healthy volunteers and tumor patients under free breathing. Participants had to provide the tracking results of 90% of the data (test set) for pre-defined point-landmarks (healthy volunteers) or for tumor segmentations (patient data). In this paper we compare the best six methods which participated in the challenge. Quantitative evaluation was performed by the organizers with respect to manual annotations. Results of all methods showed a mean tracking error ranging between 1.4 mm and 2.1 mm for 2D points, and between 2.6 mm and 4.6 mm for 3D points. Fusing all automatic results by considering the median tracking results, improved the mean error to 1.2 mm (2D) and 2.5 mm (3D). For all methods, the performance is still not comparable to human inter-rater variability, with a mean tracking error of 0.5–0.6 mm (2D) and 1.2–1.8 mm (3D). The segmentation task was fulfilled only by one participant, resulting in a Dice coefficient ranging from 76.7% to 92.3%. The CLUST database continues to be available and the online leader-board will be updated as an ongoing challenge.

## 1. Introduction

Ultrasound (US) imaging is a widely used medical imaging technique. As US has high temporal resolution and is non-ionizing, it is an appealing choice for applications which require tracking and tissue motion analysis, such as motion compensation in image-guided intervention and therapy. Conformal and minimally invasive tumor treatments, such as high intensity focused ultrasound and intensity-modulated radiation and proton therapy, deliver highly localized dose into the target tissues. Yet, the motion of the organs in the treatment region is a critical limitation. Specifically, we want to address the issue of respiratory motion in the liver (Keall *et al* 2006, Shirato *et al* 2007). Note that liver tumors are not necessarily visible in US images. Instead, other visible structures (e.g. vessels) are tracked and these tracking results are used as input surrogate data to 4D motion models to predict the tumor position (Tanner *et al* 2012, McClelland *et al* 2013).

Despite the rapid development of image-guided therapy, intervention systems and medical imaging tools, the translation into clinical practice of automated motion estimation is limited. One of the main reasons for algorithms not being integrated in clinical practice is the lack of adequate validation. Open datasets for designing and testing tracking algorithms are missing, and private datasets differ in size, image dimension and sequence length. The variations in tracking objective (full organ, anatomical landmarks, tumor) and validation strategies are additional impediments to strategy comparisons. For image-guided therapies, tracking methods should have high accuracy, robustness over the duration of the therapy and real-time capability.

Several methods have been proposed for tracking human liver structures on US sequences. Yet quantitative evaluation of tracking the human liver under free breathing was reported only by Banerjee *et al* (2015), Harris *et al* (2010), Lediju Bell *et al* (2012) and Vijayan *et al* (2014) for 3D sequences and by Cifor *et al* (2012), (2013), De Luca *et al* (2012) and De Luca *et al* (2013) for 2D sequences. Intensity-based and hybrid approaches achieved good accuracy (∼1.4 mm mean tracking error (Harris *et al* 2010) and ∼90% mean overlap ratio (Cifor *et al* 2013)). The non-rigid registration method of Vijayan *et al* (2014) estimated liver motion with an error of 1 mm (75% percentile of a root-mean squared metric over all datasets), which was lower than the inter-observer variability of 1.4 mm. More recently, a fast 3D affine block-matching algorithm with an outlier rejection strategy achieved a mean tracking error of 1.8 mm (Banerjee *et al* 2015). However, these methods were only tested off-line on short sequences (<1 min). For longer sequences (5–10 min long), a learning-based block-matching algorithm (De Luca *et al* 2013) achieved a mean tracking accuracy of $1.0\pm 0.6$ mm.

In this paper we present the outcome of the open challenge for the validation of liver motion tracking algorithms. The reported methods are selected from the ones presented at the open challenge on liver US tracking (CLUST, http://clust14.ethz.ch), held in conjunction with the 2014 international conference on medical image computing and computer assisted intervention (MICCAI 2014). The aim of CLUST was to present the current state-of-the-art in automated tracking of anatomical landmarks in the liver (vessel centers (2D), vessel bifurcations (3D) and tumor contours (2D)) and enable comparison between different methods. For the test set, the annotations of the first images were provided, which needed to be tracked over time. This paper reports the results for the full test set, while the challenge proceedings exclude 20% of the test data, which were distributed shortly before the MICCAI conference. Furthermore this publication reports results after imposing restrictions against adjusting parameters per sequence by visual inspection (which is not realistic). Thus, method parameters were either automatically determined or generally fixed for at most each US scanner and task. In addition, we investigate the inter-observer variability of the annotations, analyze various aspects influencing the tracking performance, and explore the tracking performance when fusing results.

The paper is organized as follows. In section 2 we describe the challenge data and tracking objectives. In section 3 the methods proposed by the 6 participant groups are presented. The fusion of all methods, by considering the median of their results, is also considered. The evaluation criteria are described in section 4 and the tracking results are reported and compared in section 5. Discussion and conclusions of the challenge outcome are provided in sections 6 and 7.

## 2. Materials

### 2.1. Ultrasound data

The collected database included a total of 54 US sequences of the liver of patients and volunteers under free breathing. The data were provided by 6 groups, namely the Computer Vision Laboratory, ETH Zurich, Switzerland (ETH) (De Luca *et al* 2013, Preiswerk *et al* 2014); mediri GmbH, Heidelberg, Germany (MED); Institute of Biomedical Engineering, University of Oxford, UK (OX) (Cifor *et al* 2013); Biomedical Imaging Group, Departments of Radiology and Medical Informatics, Erasmus MC, Rotterdam, The Netherlands (EMC) (Banerjee *et al* 2014); Joint Department of Physics, Institute of Cancer Research & Royal Marsden NHS Foundation Trust, London and Sutton, UK (ICR) (Lediju *et al* 2010, Lediju Bell *et al* 2012); and SINTEF Medical Technology, Image Guided Therapy, Trondheim, Norway (SMT) (Vijayan *et al* 2013).

**Figure 1.**
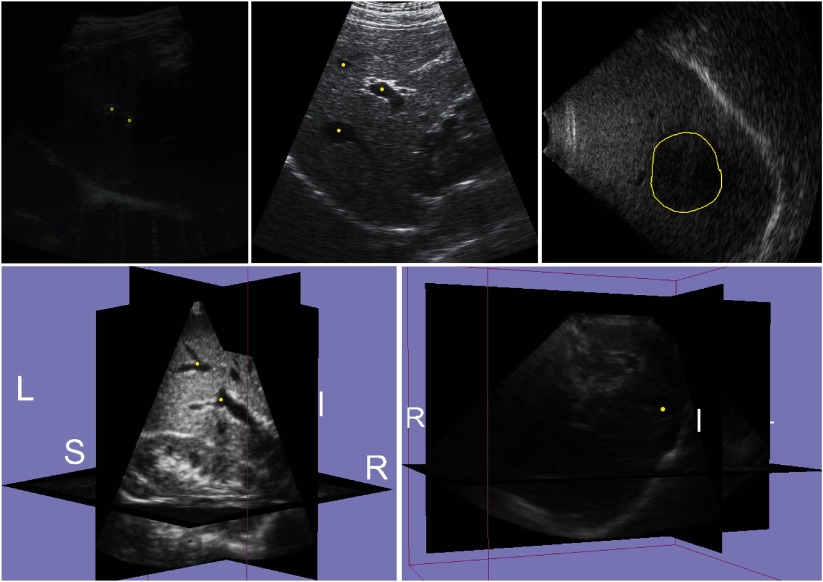
Examples of first frame *I*(0) of the training data: (top row) 2D sequences (ETH, MED, OX) and (bottom row) 3D sequences (EMC, SMT). Point-landmarks *P*_*j*_(0) and the contour of the tumor segmentation *S*_*j*_(0) are depicted in yellow.

The sequences were acquired with 6 US scanners, 7 types of transducer and different acquisition settings. An overview of the data is given in table 1. The length of the sequences ranges from 4 s to 10 min, with a temporal resolution in the range of 6–25 Hz. The dataset is divided into three subsets, according to the image dimension and annotation type. The first subset is composed of 28 2D sequences from healthy volunteers with point-landmark annotations. The second subset contains 10 2D sequences from 5 patients with segmentation annotations. The third subset consists of 16 3D sequences with point-landmark annotations from healthy volunteers. The data were anonymized and divided into a training set (10% of the sequences) and a test set (90%). For the training set annotations were released, to allow for some tuning of the tracking algorithm. For the test set, the annotations of the first images were provided. These needed to be tracked over time. Examples of the first frames and annotations are shown in figure 1.

**Table 1.** Summary of the challenge data with annotation of point-landmarks (2D sequences: ETH, MED1 and MED2; and 3D sequences: EMC, ICR and SMT) and segmentations of tumor areas (2D sequences: OX).

Sequence	Sequence info				*J*: No. annota.	No.ann. frames	Acquisition info		
	Im.size [pix]	Im.res. [mm]	*T*: No. frames	Im.rate [Hz]			Scanner	Probe	Center Freq. [MHz]
ETH-01	$264\times 313$	0.71	14516	25	1	1453	Siemens Antares	CH4-1	2.22
ETH-02	$462\times 580$	0.40	5244	16	1	525	Siemens Antares	CH4-1	2.00
ETH-03	$462\times 589$	0.36	5578	17	3	559	Siemens Antares	CH4-1	1.82
ETH-04	$472\times 565$	0.42	2620	15	1	263	Siemens Antares	CH4-1	2.22
ETH-05	$490\times 570$	0.40	3652	15	2	366	Siemens Antares	CH4-1	2.22
ETH-06	$475\times 548$	0.37	5586	17	2	560	Siemens Antares	CH4-1	1.82
ETH-07	$473\times 437$	0.28	4588	14	1	460	Siemens Antares	CH4-1	2.22
ETH-08	$466\times 562$	0.36	5574	17	2	558	Siemens Antares	CH4-1	1.82
ETH-09	$469\times 523$	0.40	5247	16	2	525	Siemens Antares	CH4-1	1.82
ETH-10	$464\times 560$	0.40	4587	15	4	460	Siemens Antares	CH4-1	1.82
ETH-11	$462\times 563$	0.42	4615	15	2	463	Siemens Antares	CH4-1	1.82
ETH-12	$478\times 552$	0.45	4284	14	2	429	Siemens Antares	CH4-1	2.22
MED-01	$512\times 512$	0.41	2470	20	3	248	DiPhAs Fraunhofer	VermonCLA	5.5
MED-02	$512\times 512$	0.41	2478	20	3	248	DiPhAs Fraunhofer	VermonCLA	5.5
MED-03	$512\times 512$	0.41	2456	20	4	246	DiPhAs Fraunhofer	VermonCLA	5.5
MED-04	$512\times 512$	0.41	2455	20	3	246	DiPhAs Fraunhofer	VermonCLA	5.5
MED-05	$512\times 512$	0.41	2458	20	3	246	DiPhAs Fraunhofer	VermonCLA	5.5
MED-06	$512\times 512$	0.41	2443	20	3	245	DiPhAs Fraunhofer	VermonCLA	5.5
MED-07	$512\times 512$	0.41	2450	20	3	246	DiPhAs Fraunhofer	VermonCLA	5.5
MED-08	$512\times 512$	0.41	2442	20	2	245	DiPhAs Fraunhofer	VermonCLA	5.5
MED-09	$512\times 512$	0.41	2436	20	5	244	DiPhAs Fraunhofer	VermonCLA	5.5
MED-10	$512\times 512$	0.41	2427	20	4	243	DiPhAs Fraunhofer	VermonCLA	5.5
MED-11	$512\times 512$	0.41	2424	20	3	243	DiPhAs Fraunhofer	VermonCLA	5.5
MED-12	$512\times 512$	0.41	2450	20	3	246	DiPhAs Fraunhofer	VermonCLA	5.5
MED-13	$524\times 591$	0.35	3304	11	3	331	Zonare z.one	C4-1	4.0
MED-14	$524\times 591$	0.35	3304	11	3	331	Zonare z.one	C4-1	4.0
MED-15	$524\times 591$	0.35	3304	11	1	331	Zonare z.one	C4-1	4.0
MED-16	$524\times 591$	0.35	3304	11	2	331	Zonare z.one	C4-1	4.0
OX-01	$416\times 528$	0.30	71	12	1	71	Zonare z.one	P4-1	3.6
OX-02	$336\times 448$	0.40	82	12	1	82	Zonare z.one	P4-1	3.6
OX-03	$416\times 528$	0.38	82	12	1	82	Zonare z.one	P4-1	3.4
OX-04	$336\times 448$	0.36	51	14.5	1	51	Zonare z.one	C6-2	4.4
OX-05	$337\times 448$	0.46	101	11.7	1	101	Zonare z.one	C6-2	3.8
OX-06	$337\times 449$	0.55	76	11	1	76	Zonare z.one	C6-2	3.8
OX-07	$338\times 450$	0.50	63	10	2	63	Zonare z.one	C6-2	3.8
OX-08	$337\times 448$	0.46	105	11	1	105	Zonare z.one	C6-2	3.8
OX-09	$338\times 450$	0.50	98	10	2	98	Zonare z.one	C6-2	3.8
OX-10	$337\times 449$	0.55	92	11	1	92	Zonare z.one	C6-2	3.8
EMC-01	$192\times 246\times 117$	$1.14\times 0.59\times 1.19$	79	6	1	8	iU22	X6-1	3.2
EMC-02	$192\times 246\times 117$	$1.14\times 0.59\times 1.19$	54	6	4	6	iU22	X6-1	3.2
EMC-03	$192\times 246\times 117$	$1.14\times 0.59\times 1.19$	159	6	1	16	iU22	X6-1	3.2
EMC-04	$192\times 246\times 117$	$1.14\times 0.59\times 1.19$	140	6	1	15	iU22	X6-1	3.2
EMC-05	$192\times 246\times 117$	$1.14\times 0.59\times 1.19$	147	6	1	15	iU22	X6-1	3.2
ICR-01	$480\times 120\times 120$	$0.31\times 0.51\times 0.67$	141	24	1	15	Siemens SC2000	4Z1c	2.8
ICR-02	$480\times 120\times 120$	$0.31\times 0.51\times 0.67$	141	24	1	20	Siemens SC2000	4Z1c	2.8
SMT-01	$227\times 227\times 229$	0.70	97	8	3	96	GE E9	4V-D	2.5
SMT-02	$227\times 227\times 229$	0.70	96	8	3	92-93	GE E9	4V-D	2.5
SMT-03	$227\times 227\times 229$	0.70	96	8	2	45-96	GE E9	4V-D	2.5
SMT-04	$227\times 227\times 229$	0.70	97	8	1	96	GE E9	4V-D	2.5
SMT-05	$227\times 227\times 229$	0.70	96	8	2	64-96	GE E9	4V-D	2.5
SMT-06	$227\times 227\times 229$	0.70	97	8	3	49-96	GE E9	4V-D	2.5
SMT-07	$227\times 227\times 229$	0.70	97	8	2	95	GE E9	4V-D	2.5
SMT-08	$227\times 227\times 229$	0.70	97	8	3	96	GE E9	4V-D	2.5
SMT-09	$227\times 227\times 229$	0.70	97	8	3	96	GE E9	4V-D	2.5

*Note*: The test set is listed in **black** font. The training sequences, for which all available annotations were provided, are highlighted in **red**.

Given a sequence of *T* images *I*(*t*), $I\left(t,\mathbf{x}\right)$ denotes the intensity (or brightness) of image *I*(*t*) at position $\mathbf{x}$ at frame *t*, with $t=0,\ldots ,T-1$ and $\mathbf{x}={{\left({{x}_{1}},\ldots ,{{x}_{D}}\right)}^{T}}\in {{\mathbb{R}}^{D}}$ (*D* = 2, 3). Depending on the subset, the tracking objective was to compute either the position of *J* point-landmarks ${{P}_{j}}(t)\in {{\mathbb{R}}^{D}}$ or the segmentation of *J* tumor areas ${{S}_{j}}\left(t,\mathbf{x}\right)\in \left[0,1\right]$ in each image, with $j=1,\ldots ,J$ and $J\in \left\{1,\ldots ,5\right\}$ in this challenge. The displacement of landmark *j* at time *t* is denoted as ${{\mathbf{d}}_{j}}(t)={{P}_{j}}(t)-{{P}_{j}}(0)$. For all sequences, annotations of the first frame (*P*_*j*_(0) or *S*_*j*_(0)) were provided.

## 3. Methods

The challenge raised interest from 55 individuals worldwide. All of them successfully downloaded the data. Only 8 from the downloaders submitted their results to CLUST14. After the withdrawal of one of the groups, a total of 7 papers were accepted to the MICCAI workshop. In this paper we included the 6 contributions with the highest mean accuracy, namely (in alphabetical order of the abbreviations) from Konica Minolta Inc., Osaka, Japan (KM); Fraunhofer MEVIS, Lübeck, Germany (MEVIS); Fraunhofer MEVIS, Bremen and Fraunhofer FOKUS, Berlin, Germany (MEVIS + FOKUS); Fraunhofer MEVIS, Bremen and Mediri GmbH, Heidelberg, Germany (MEVIS + MED); Philips Research, Suresnes, France (PhR); and Technische Universität München, Germany (TUM).

In the following we briefly describe each algorithm. An overview of the methods’ main features is presented in table 2. All 6 presented methods were tested on 2D landmark tracking. Of these, 3 were extended to 3D tracking, while only one submission covered all the challenge tasks. A more detailed description of each method can be found in the workshop proceedings^15^15The challenge proceedings are available at http://clust14.ethz.ch/clust2014.html.

**Table 2.** Summary of the main features of the evaluated tracking methods: the tracking **Task** namely 2D and 3D point-landmark (**2D p.** and **3D p.** respectively) and 2D tumor segmentation (**2D s.**); the key components of the tracking algorithm (**Keywords**).

Participant	Task			Keywords	Real-time
	2D p.	2D s.	3D p.		
KM	✓	✗	✗	block matching, NCC, local translation, exhaustive search	✗
MEVIS	✓	✗	✗	variational, large moving ROI, SSD & NGF, curvature regularizer	✗
MEVIS + FOKUS	✓	✗	✓	optical flow, histogram equalization, 30% downsampling, polynomial expansion, bilateral filtering, outlier detection, 2 orthogonal slices	✓
MEVIS + MED	✓	✗	✓	Bayesian approach, particle filter, intensity difference, local translation	✓
PhR	✓	✓	✓	sparse Demons, ROI, SSD, fluid regularizer, gradient descent, drift prevention strategy	✓
TUM	✓	✗	✗	kernel-based, intensity distribution similarity, adaptive ellipsoidal target descriptor, local affine, failure recovery strategy	✓

*Note*: **Real-time** capability was assessed w.r.t. the average frame rate of 20 Hz (50 ms) for 2D sequences and 8 Hz (125 ms) for 3D sequences.

### 3.1. KM: template matching

Kondo, Konica Minolta Inc. (KM), developed a multiple-template matching method, based assuming that all regions of interest (ROIs) from the same image move along almost the same direction, and the motion has high periodicity due to breathing. Similar to De Luca *et al* (2013) and Matthews *et al* (2004), templates are selected from a plurality of recent frames to exploit the highly periodicity. The method consists of five steps:

**Step 1: selection of global and long-term templates.** From the first image *I*(0) two templates (i.e. unchanging subimages) are selected: a global template ${{T}_{\text{G}}}\left(\mathbf{x}\right)$, which is used to determine the motion on the entire frame and is defined by the largest rectangle which can be inscribed in the US image; and a long-term template ${{T}_{L,j}}\left(\mathbf{x}\right)$, which is a squared ROI around the annotated point *P*_*j*_(0), whose size is based on minimizing the variance of the pixel values inside the ROI. The set of pixel coordinates included in ${{T}_{L,j}}\left(\mathbf{x}\right)$ and ${{T}_{g}}\left(\mathbf{x}\right)$ are denoted as *B*_*L*,*j*_ and ${{B}_{\text{G}}}$, respectively.

**Step 2: global motion estimation.** For each image *I*(*t*), *t*  >  0, the global displacement ${{\mathbf{d}}_{\text{G}}}(t)$, of point *P*_*j*_(0), is estimated by maximizing the normalized cross-correlation (NCC) over ${{T}_{\text{G}}}\left(\mathbf{x}\right)$ using exhaustive search:

1 \begin{eqnarray*}{{\mathbf{d}}_{\text{G}}}(t)=\text{argma}{{\text{x}}_{\mathbf{d}}}\text{NC}{{\text{C}}_{\text{G}}}\left(t,\mathbf{d}\right),\end{eqnarray*}

where

2 \begin{eqnarray*}\text{NC}{{\text{C}}_{k}}\left(t,\mathbf{d}\right)=\frac{{\sum}_{\mathbf{x}\in {{B}_{k}}}{{T}_{k}}\left(\mathbf{x}\right)I\left(t,\mathbf{x}+\mathbf{d}\right)}{\sqrt{{\sum}_{\mathbf{x}\in {{B}_{k}}}{{T}_{k}}{{\left(\mathbf{x}\right)}^{2}}\cdot {\sum}_{\mathbf{x}\in {{B}_{k}}}I{{\left(t,\mathbf{x}+\mathbf{d}\right)}^{2}}}}.\end{eqnarray*}

**Step 3: long-term motion estimation.** Similarly the long-term motion component ${{\mathbf{d}}_{L,j}}(t)$ over ${{T}_{L,j}}\left(\mathbf{x}\right)$ is computed as

3 \begin{eqnarray*}{{\mathbf{d}}_{L,j}}(t)=\text{argma}{{\text{x}}_{\mathbf{d}\in {{S}_{L}}}}\text{NC}{{\text{C}}_{L,j}}\left(t,\mathbf{d}\right),\end{eqnarray*}

where *S*_*L*_ is the set of tested pixel displacements. It depends on the global motion estimation (Step2) as follows: if $\text{NC}{{\text{C}}_{\text{G}}}\left(t,{{\mathbf{d}}_{\text{G}}}(t)\right)&gt;0.95$ then ${{S}_{L}}={{\mathbf{d}}_{\text{G}}}(t)\pm 7$ pixels, otherwise *S*_*L*_=±15 pixels. The motion vectors ${{\mathbf{d}}_{L,j}}(t)$ and $\text{NC}{{\text{C}}_{L,j}}\left(t,{{\mathbf{d}}_{L,j}}(t)\right)$ are stored in the short-term buffer.

**Step 4: short-term motion estimation.** Firstly, the cycle length *c* is estimated from the past tracking results. Then, two short-term templates for the *t*-th frame are selected: the ROI with the maximum $\text{NC}{{\text{C}}_{L,j}}\left({{t}_{S1}},{{\mathbf{d}}_{L,j}}\left({{t}_{S1}}\right)\right)$, with ${{t}_{S1}}\in \left[t-c,t-1\right]$; and the ROI with the minimum $\parallel {{\mathbf{d}}_{L,j}}\left({{t}_{S2}}\right)-{{\mathbf{d}}_{L,j}}(t-1)\parallel $ in ${{t}_{S2}}\in \left[t-3c/2,t-c/2\right]$. Principal component analysis (PCA) is applied to the 2D trajectory of the tracking positions, and the motion estimation is performed only in the first principal direction. The resulting ${{\mathbf{d}}_{S,j}}(t)$ is determined by providing the maximum NCC for these templates ($\text{NC}{{\text{C}}_{S,j}}\left(t,\mathbf{d}\right)$).

**Step 5: final tracking.** The final motion estimation ${{\mathbf{d}}_{j}}(t)$ is given by:

4 \begin{equation*} {\bf d}_j(t) = \left\{ \begin{array}{l l} {\bf d}_{L,j}(t) \quad {\rm if}~{\rm NCC}_{L,j}(t,{\bf d}_{L,j}(t))\geqslant \alpha ~{\rm NCC}_{S,j}(t,{\bf d}_{S,j}(t)) \\ {\bf d}_{S,j}(t) \quad {\rm otherwise} \end{array}\right\}, \end{equation*}

where $\alpha =0.95$ is a weight that prioritizes the long-term motion estimation.

**Run-time.** This method was implemented in C++ using OpenCV and OpenMP on a computer with Intel Core i7 3.3 GHz GPU, 6 cores and 64 GB memory. The average processing time was approximately 84 ms per frame, which can be improved by optimizing the motion estimation routine, currently based on an OpenCV function.

### 3.2. MEVIS: variational real-time registration

König *et al* Fraunhofer MEVIS Project Group Image Registration, Lübeck, Germany, (MEVIS) proposed a novel scheme for point tracking in long US sequences, based on an efficient, state-of-the-art variational non-linear registration method (Modersitzki 2009, König and Rühaak 2014). It is extended by a moving window scheme with additional fallback strategy to minimize tracking failures.

**Registration algorithm.** Transformation $\mathbf{T}$ between images *A* and *B* was determined by a variational approach with the objective function $\mathcal{J}\left(\mathbf{T}\right)={{\mathcal{D}}_{\text{SSD}}}\left(A,\mathbf{T}(B)\right)+\beta {{\mathcal{D}}_{\text{NGF}}}\left(A,\mathbf{T}(B)\right)+\alpha \mathcal{S}\left(\mathbf{T}\right)$, where ${{\mathcal{D}}_{\text{SSD}}}$ is the sum of squared differences (SSD), ${{\mathcal{D}}_{\text{NGF}}}$ is the edge-based normalized gradient fields (NGF) (Haber and Modersitzki 2006), and $\mathcal{S}$ is the second order curvature regularizer (Fischer and Modersitzki 2003). See König *et al* (2014), König and Rühaak (2014) and Modersitzki (2009) for details on the numerical optimization.

**Figure 2.**
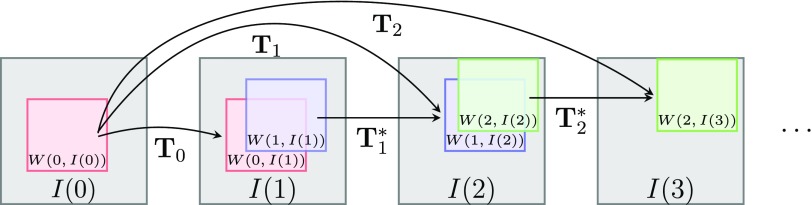
Tracking scheme. First *W*(*t*  −  1, *I* (*t*)) is registered to *W*(0, *I*(0)) providing ${{\mathbf{T}}_{t}}$. If this registration fails, *W*(*t*  −  1, *I*(*t*)) is registered to corresponding window of previous frame ($\mathbf{T}_{t}^{*}$).

**Tracking scheme.** The non-linear registration algorithm is embedded in a tracking framework. By calculating registrations of moving windows on each frame, ${{P}_{j}}(0)\in {{\mathbb{R}}^{2}}$ is tracked over time, see figure 2.

$W\left(\tau ,I(t)\right):{{\mathbb{R}}^{M\times N}}\to {{\mathbb{R}}^{{{w}_{1}}\times {{w}_{2}}}},\tau ,t=0,\ldots ,T-1$ is defined as a window of $I(t)\in {{\mathbb{R}}^{M\times N}}$ with extent ${{w}_{1}},{{w}_{2}}\in \mathbb{N},{{w}_{1}}\leqslant M,{{w}_{2}}\leqslant N$ and center position ${{C}_{j}}(\tau )\in {{\mathbb{R}}^{2}}$. First, window *W*(0,*I*(0)) with center *C*_*j*_(0)=*P*_*j*_(0) is chosen. Then *W*(0,*I*(1)) is registered to *W*(0,*I*(0)) and *P*_*j*_(0) is transformed by ${{\mathbf{T}}_{0}}(({{P}_{j}}(0))$ to get *P*_*j*_(1). Using *C*_*j*_(*t*) = *P*_*j*_(*t*) as the center of a new window, this process is then repeated for all frames.

As a safeguard, the original intensity at position *P*_*j*_(0) is compared to the current intensity at *P*_*j*_(*t*). If $\mid I\left({{P}_{j}}(0),0\right)-I\left({{P}_{j}}(t),t\right)\mid &gt;I\left({{P}_{j}}(0),0\right)\theta +5$, the registration paradigm is switched and window *W*(*t*  −  1,*I*(*t*)) is registered to *W*(*t*  −  1,*I*(*t*  −  1)), yielding $\mathbf{T}_{t-1}^{*}$.

**Parametrization.** Parameters for optimization, deformation resolution ($17\times 17$) and multi-level scheme (2 levels) were kept constant. The window size was 50 mm in each dimension. Weight $\beta $ is automatically determined before each registration as $\beta ={{\mathcal{D}}_{\text{SSD}}}(A,B)/{{\mathcal{D}}_{\text{NGF}}}(A,B)$. The parameters $\alpha $ and $\eta $ (edge parameter of NGF) were manually chosen per scanner (ETH datasets: $\alpha =10,\eta =20$, MED1: $\alpha =5,\eta =5$, MED2: $\alpha =100,\eta =2$). The threshold was $\theta =0.5$ for ETH and MED2, and $\theta =0.75$ for MED1.

**Run-time.** The algorithm achieved close to real-time performance in all cases (23–90 ms per frame), exceeding acquisition rate in 48% of all cases, computed on a three year old Intel i7-2600 PC with 3.40 GHz. Thus real-time performance is easily within reach when using recent hardware.

### 3.3. MEVIS + FOKUS: high performance optical flow

The method of Lübke, Fraunhofer MEVIS, Bremen, and Grozea, Fraunhofer FOKUS, Berlin, Germany, (MEVIS + FOKUS) is based on a two-frame motion estimation by an optical flow approach using polynomial expansion (Farnebäck 2003) and builds on the OpenCV function *calcOpticalFlowFarneback*. It yields a motion vector field for the entire frame which allows to track multiple points in parallel without any computational overhead. The method consists of approximating images locally with quadratic polynomials and then obtaining the dense displacement field by inferring the local displacement analytically. This is based on the coefficients of the fitted polynomial surfaces, with smoothing coming from the assumption of a global parametrized displacement model. In contrast to the original method, the OpenCV implementation did not provide a certainty for each pixel. This reduced to some extent the accuracy when the tracked point approached repeatedly the border of the acquisition region.

Real-time capability is achieved by downscaling the images before tracking and filtering, and subsequent upscaling of the result to the original resolution. A fixed downscaling factor to 30% has been chosen to match the computation time with the frame-rate. Fixed-parameter bilateral filtering and histogram equalization are used to obtain more stable results. Outliers, which are defined as motion vector component changes greater than 12 pixels, are discarded and the previous positions are used instead. Linear fitting (sliding window) is used to smooth the trajectory for each dimension to eliminate high-frequency motion.

This method was applied to 2D and 3D datasets. In the latter case it is in fact a 2.5D method, as we are evaluating the motion in two orthogonal slices intersecting at the manual annotation. This yields two separate tracking results with redundant information on the intersection. The method is sensitive to out-of-plane-motion as any 2D tracking method, since no adjustment based on the combined information has yet been done.

Another approach was proposed by this group and applied on 2D datasets, see the CLUST14 proceedings for details (Lübke and Grozea 2014). It was based on patch-matching, maximizing NCC, random sampling, explicit masking to improve accuracy at the border, and GPU implementation. This approach was not included in this paper, as it was on average slower (84%) and had a similar mean accuracy (3% better) as the group’s included method.

**Run-time.** The method was implemented using the Mevislab software (MeVis Medical Solutions, Bremen, Germany), Python, OpenCV and Numpy. The run-time was measured on a Intel Core i7-4770k with 32 GB RAM. The average run-time for all 2D points is 40 ms. As the 3D tracking is performed on two orthogonal slices, the average processing time per volume of 61 ms is slightly higher than in the 2D datasets but still below the 3D images frame-rate (real-time), except for the ICR data subset with temporal resolution of 24 Hz.

### 3.4. MEVIS + MED: Bayesian tracking

Rothlübbers *et al* Fraunhofer MEVIS, Bremen, Germany, (MEVIS + MED) proposed a Bayesian approach for tracking point landmarks in 2D and 3D US sequences.

**Particle Filter.** A particle filter based algorithm (Isard and Blake 1996, Arulampalam *et al* 2002, Zhang *et al* 2010) is used to track *P*_*j*_(0) in a 2D or 3D sequence. A small region around target *P*_*j*_(0) is considered to translate through the sequence, yielding a *d* = 2,3 dimensional state space for 2D and 3D translations respectively. The state of the target is represented by a probability density function in state space, approximated by a fixed size set of *N* particles. Each particle *n* holds the state ${{\mathbf{s}}_{n}}(t)=\left\{s_{n}^{d}(t)\right\}$ of a position hypothesis and the associated weight *w*_*n*_(*t*), normalized by $\underset{n}{\sum}\,{{w}_{n}}(t)=1$, for $n=1,\ldots ,N$. With each tracking step ${{\mathbf{s}}_{n}}$ and *w*_*n*_ are updated by estimations and observations, respectively.

**Figure 3.**
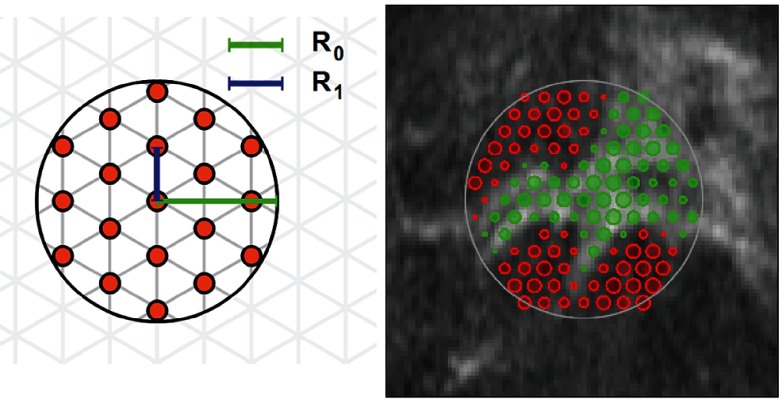
Initialization: (Left) Within radius *R*_0_ of a given position, points on a local triangular grid with grid constant *R*_1_ are chosen. (Right) Example of point weights ($p_{i}^{\text{brt}}-p_{i}^{\text{drk}}$, see text) in a first frame: area indicates value and color encodes sign (red: negative, green: positive).

**Target Description.** The target region is described by an under-sampled grid, characterized by radius *R*_0_ around *P*_*j*_(0) and grid constant *R*_1_ (see figure 3), resulting in ${{N}_{\text{P}}}$ grid points ${{\mathbf{r}}_{i}}$, $i=1,\ldots ,{{N}_{\text{P}}}$. Each ${{\mathbf{r}}_{j}}$ has associated information for belonging to the bright $p_{i}^{\text{brt}}$ and dark $p_{i}^{\text{drk}}$ part of the region (${\sum}^{}p_{i}^{\text{brt}}=1$,${\sum}^{}p_{i}^{\text{drk}}=1$) derived from the intensities in the initial frame ($p_{i}^{\text{brt}}\propto \left({{b}_{i}}-{{b}_{\text{min}}}\right)$, $p_{i}^{\text{drk}}\propto \left({{b}_{\text{max}}}-{{b}_{i}}\right)$) where *b*_max_ and ${{b}_{\text{min}}}$ are the maximal and minimal intensity among all ${{b}_{i}}\in I\left(0,{{\mathbf{r}}_{i}}\right)$.

**Estimation.** States are updated by re-sampling (Isard and Blake 1996) and application of stochastic drift and diffusion (equation (5)), taking into account the direct predecessor state (Markov property):

5 \begin{eqnarray*}s_{n}^{d}(t+1)={{\langle s_{n}^{d}(t)\rangle}_{n}}+S_{0}^{d}\left[s_{n}^{d}(t)-{{\langle s_{n}^{d}(t)\rangle}_{n}}\right]+S_{1}^{d}\eta ,\end{eqnarray*}

where $S_{0}^{d}$ determines the amount of drift towards the mean state ${{\langle s_{n}^{d}(t)\rangle}_{n}}$ and $S_{1}^{d}$ scales the diffusion by normally-distributed Gaussian noise ($\eta $).

**Observation.** Weights are updated from ${{\mathbf{s}}_{n}}(t+1)$ and the incoming image *I*(*t*+1) using the translation matrix ${{\mathbf{T}}_{{{\mathbf{s}}_{n}}(t+1)}}$ to transform points ${{\mathbf{r}}_{i}}$ into *I*(*t*+1) by:

6 \begin{eqnarray*}w_{n}^{\prime}(t+1)=\underset{i=1}{\overset{{{N}_{\text{P}}}}{\sum}}\,\left[p_{i}^{\text{brt}}-p_{i}^{\text{drk}}\right]\cdot I\left(t+1,{{\mathbf{T}}_{{{\mathbf{s}}_{n}}(t+1)}}\left({{\mathbf{r}}_{i}}\right)\right).\end{eqnarray*}

When coinciding with bright image areas, bright grid points increase the weight while dark grid points decrease it. The final weighting function is

7 \begin{eqnarray*}{{w}_{n}}(t+1)=\Theta\left(w_{n}^{\prime}(t+1)\right)w_{n}^{\prime 2}(t+1),\end{eqnarray*}

with Heaviside function $\Theta(\cdot )$ to set negative weights to zero, and with squaring of weights as this improved performance. The tracking result is computed to be the centroid of the target model, transformed into the image by the weighted mean sample. The tracking step was repeated *M* times to allow the distribution’s mean to settle, leading to reduced lag.

**Execution.** Per target, *R*_0_ and *R*_1_ were adapted to fit the given target scenario and resolution of *I*(0), ranging from small speckle-like features to large dark vessels. Typical values for 2D are ${{R}_{0}}\approx 25$, ${{R}_{1}}\approx 4$ pixels resulting in ${{N}_{\text{P}}}\approx 130$. The other parameters were set to *N* = 400, $S_{0}^{d}=1$, $S_{1}^{d}=2\left(\text{ETH}\right),3\left(\text{MED}1\right),1.2\left(\text{MED}2\right)$, and *M* = 2. For the 3D sets with generally lower resolution, ${{R}_{0}}\approx 13$, ${{R}_{1}}\approx 2$ voxels and ${{N}_{\text{P}}}\in \left[300,5000\right]$ was increased due to the additional third dimension. For 3D, parameters were set to *N* = 200, $S_{0}^{d}=1$, $S_{1}^{d}=1.5\left(\text{EMC},\text{ICR},\text{SMT}\right)$, and *M* = 4.

**Run-time.** With the semi-manual initialization, the algorithm performs very efficiently, with an average run-time of $1.25\pm 0.84$ ms per 2D frame and $46.76\pm 84.50$ ms per 3D volume. The core source code is written in C++ and integrated into MeVisLab (MeVis Medical Solutions, Bremen, Germany) for high level evaluation routines using Python scripts. The code was executed single threaded on a Windows 7 machine with an Intel Core i7-2600 CPU @ 3.4 GHz and 32 GB RAM.

### 3.5. PhR—sparse demons

Somphone *et al* Medisys Lab, Philips Research, Suresnes, France, (PhR) based their method on the Sparse Demons framework, where a dense displacement field is found by minimizing an energy *E* defined only on a small number $\mathcal{P}$ of points of interest $\left\{{{\mathbf{x}}_{i}}\,\mid \,i\in \mathcal{P}\right\}$:

8 \begin{eqnarray*}E=\underset{i\in \mathcal{P}}{\sum}\,{\int}_{\Omega}\delta \left(\mathbf{x}-{{\mathbf{x}}_{i}}\right)\mathcal{D}\left[R\left(\mathbf{x}\right)-T\left(\mathbf{x}+\mathbf{u}\left(\mathbf{x}\right)\right)\right]\text{d}\mathbf{x},\end{eqnarray*}

where *R* and *T* are the reference and template images respectively, $\Omega$ is the image domain and $\delta $ is the Dirac function. The dissimilarity between the images is measured by $\mathcal{D}$ (Somphone *et al* 2013). Regularization is based on filtering the fluid-like field $\mathbf{v}$ with a Gaussian kernel ${{\omega}_{\sigma}}$ of scale $\sigma =30$ mm (Mory *et al* 2012):

9 \begin{eqnarray*}\mathbf{u}\left(\mathbf{x}\right)=\left[{{\omega}_{\sigma}}*\mathbf{v}\right]\left(\mathbf{x}\right)={\int}_{\Omega}{{\omega}_{\sigma}}\left(\mathbf{x}-\mathbf{y}\right)\mathbf{v}\left(\mathbf{y}\right)\text{d}\mathbf{y},\text{where}\ {{\omega}_{\sigma}}\left(\mathbf{x}\right)=\frac{1}{2\pi {{\sigma}^{2}}}e\frac{\parallel \mathbf{x}\parallel}{2{{\sigma}^{2}}}.\end{eqnarray*}

Minimizing *E* w.r.t. $\mathbf{v}$ is done by gradient descent. Calculus of variations results in the following evolution equation:

10 \begin{eqnarray*}\frac{\partial \mathbf{v}}{\partial t}=-{{\nabla}_{\mathbf{v}}}E=-{{\omega}_{\sigma}}*\left(\underset{i\in \mathcal{P}}{\sum}\,{{\delta}_{i}}{{\nabla}_{\mathbf{u}}}E\right),\end{eqnarray*}

where ${{\delta}_{i}}\left(\mathbf{x}\right)=\delta \left(\mathbf{x}-{{\mathbf{x}}_{i}}\right)$ and ${{\nabla}_{\mathbf{u}}}E$ is the dense gradient of *E* w.r.t. $\mathbf{u}$:

11 \begin{eqnarray*}{{\nabla}_{\mathbf{u}}}E\left(\mathbf{x}\right)=-{{\mathcal{D}}^{\prime}}\left[I\left(0,\mathbf{x}\right)-I\left(t,\mathbf{x}+\mathbf{u}\left(\mathbf{x}\right)\right)\right]\nabla T\left(\mathbf{x}+\mathbf{u}\left(\mathbf{x}\right)\right).\end{eqnarray*}

The subsequent algorithm has similarities with the Demons algorithm (Thirion 1998, Mansi *et al* 2011). Its computational complexity is however lower since image forces are only computed at points ${{\mathbf{x}}_{i}}$.

**Algorithm 1.** Sparse Demons—Gradient Descent

1: $k\leftarrow 0$;
2: ${{\mathbf{v}}^{0}}\leftarrow 0$;
3: **repeat**
4: $\text{Compute}\ {{u}^{k}}={{\omega}_{\sigma}}*{{\mathbf{v}}^{k}}$;
5: **for all** *x*_*i*_ **do**
6: $\text{Interpolate}\ T\left(x+{{u}^{k}}\left({{x}_{i}}\right)\right)\text{and}\ \nabla T\left(x+{{u}^{k}}\left({{x}_{i}}\right)\right)$;
7: $\text{Compute}{{\nabla}_{{{u}^{k}}}}E\left({{x}_{i}}\right)$; ⊳ according to (11)
8: **end for**
9: $\text{Smooth result to get incremental update}\ \delta {{\mathbf{v}}^{k}}=-{{\omega}_{\sigma}}*\left({\sum}_{i\in \mathcal{P}}{{\delta}_{i}}{{\nabla}_{{{u}^{k}}}}E\right)$;
10: $\text{Update}\ {{\mathbf{v}}^{k+1}}={{\mathbf{v}}^{k}}+\delta t\delta {{\mathbf{v}}^{k}}$;
11: *k* = *k*+1;
12: **until** steady state

**2D point tracking.** $\mathcal{D}(x)={{x}^{2}}/2$ is chosen as dissimilarity measure. The reference points of interest are chosen in the neighborhood of the landmarks, based on having a high amplitude of the image gradient. The tracking strategy consists of two phases. In the initial (*t*  −  1)-to-*t* strategy for $t\in \left[0,100\right]$, *R* = *I*(*t*  −  1) and *T* = *I*(*t*), and a mean reference patch (of size $30\times 30$ ixels^2^) is built around *P*_*j*_(0) from the patches centered on the tracked landmark positions. To prevent error accumulation, the 0-to-*t* patch tracking is used from *t*  >  100.

**3D point tracking.** Reference points ${{\mathbf{x}}_{i}}$ are chosen in the neighborhood of the landmark in a $80\times 80\times 80$ mm^3^ region, regularly spaced by 10 mm. The baseline tracking follows the (*t*  −  1)-to-*t* scheme. To prevent drifting, a 0-to-*t* tracking is enabled if the difference between the histograms of *I*(*t*) and *I*(0) is lower than 20% of the histogram difference between *I*(1) and *I*(0), or landmarks positions are closer than 1.8 mm to *P*_*j*_(0).

**2D segmentation tracking.** The entropy of the difference between the reference and the transformed template is used as dissimilarity measure, since the OX sequences display large intensity changes between frames. The points of interest ${{\mathbf{x}}_{i}}$ are selected in the neighborhood of the segmentation boundary, again based on the image gradient amplitude. As the OX sequences are short, only the (*t*  −  1)-to-*t* scheme is used.

**Run-time** The average processing time of 25 ms for 2D and 100 ms for 3D was obtained on a multithreaded PC platform. Yet, the method was not specifically optimized for run-time.

### 3.6. TUM: kernel-based tracking

Benz *et al* Technische Universität München, Germany, (TUM) proposed a kernel-based US tracking method. The target model $q={{\left\{{{q}_{u}}\right\}}_{u=1,\ldots ,m}}$ centered at *I*(0,*P*_*j*_(0)) and the target candidate model $p\left(\mathbf{x}\right)={{\left\{{{p}_{u}}\right\}}_{u=1,\ldots ,m}}$ centered at $I\left(t,\mathbf{x}\right)$ are represented by normalized weighted intensity histograms, with *m* = 32 number of histogram bins. Each pixel contribution to a histogram bin *u* is weighted based on the radially symmetric Epanechnikov kernel (Epanechnikov 1969), which assigns smaller weights to pixel locations farther away from the center.

In each frame *I*(*t*) the goal is to find $p\left(\mathbf{x}\right)$ that best matches *q*. The discrete Bhattacharyya coefficient (Comaniciu *et al* 2003) $\rho \left(\mathbf{x}\right)={{\sum}_{u=1}^{m}}\,\sqrt{{{p}_{u}}\left(\mathbf{x}\right){{q}_{u}}}$ is used as similarity measure between *q* and $p\left(\mathbf{x}\right)$. In each frame *t* the procedure to find the location $\mathbf{\hat{x}}$ that maximizes $\rho \left(\mathbf{x}\right)$ is started at location ${{\mathbf{\hat{x}}}_{0}}$, which initially is set to the previous solution ${{\mathbf{\hat{x}}}_{t-1}}$. After linearization through a Taylor series expansion around $\widehat{{{\mathbf{x}}_{0}}}$, $\rho \left(\mathbf{x}\right)$ can be maximized using the mean shift procedure (Fukunaga and Hostetler 1975). When using the Epanechnikov kernel, the mean shift iteration step to move the kernel center position from ${{\mathbf{\hat{x}}}_{0}}$ to its new position ${{\mathbf{\hat{x}}}_{1}}$ is

12 \begin{eqnarray*}{{\mathbf{\hat{x}}}_{1}}=\frac{\underset{i=1}{\overset{{{n}_{h}}}{\sum}}\,{{x}_{i}}{{w}_{i}}}{\underset{i=1}{\overset{{{n}_{h}}}{\sum}}\,{{w}_{i}}}.\end{eqnarray*}

The weight *w*_*i*_ for each pixel depends on a comparison of the histogram bins of *q*_*u*_ and ${{p}_{u}}\left(\mathbf{x}\right)$ that intensity $I\left(t,{{\mathbf{x}}_{i}}\right)$ falls into:

13 \begin{eqnarray*}{{w}_{i}}=\underset{u=1}{\overset{m}{\sum}}\,\delta \left[b\left(I\left(t,{{\mathbf{x}}_{i}}\right)\right)-u\right]\sqrt{\frac{{{q}_{u}}}{{{p}_{u}}\left(\mathbf{x}\right)}},\end{eqnarray*}

where $\delta $ is the Kronecker delta function and $b\left(I\left(t,{{\mathbf{x}}_{i}}\right)\right)$ a binning function that maps intensity $I\left(t,{{\mathbf{x}}_{i}}\right)$ to a histogram bin number. After each mean shift iteration, convergence is checked (number of iterations  >20 or mean shift vector length  <0.05 pixels) and if met, ${{\mathbf{\hat{x}}}_{t}}$ is set to ${{\mathbf{\hat{x}}}_{1}}$, otherwise ${{\mathbf{\hat{x}}}_{0}}$ is set to ${{\mathbf{\hat{x}}}_{1}}$ and the mean shift procedure is repeated.

This method incorporates modifications proposed by Ning *et al* (2012) to make it adaptive to scale and orientation. First the target’s scale is estimated based on the sum of the weights of all pixels in the search region and adjusted by the current Bhattacharyya coefficient. The estimated area is then used to adjust an ellipsoidal target descriptor to match the width, height and orientation of the ellipsoidal target descriptor, which is manually initialized in *I*(0). Between two adjacent frames the kernel size is enlarged by parameter $\Delta d$, which is set to 1 and 3 for all ETH and MED sequences respectively.

Assuming motion periodicity, two failure recovery strategies are employed. First, if the Bhattacharyya coefficient $\rho $ drops below 0.8, the found target position is discarded and tracking is repeated using the target search area defined in *I*(0). Second, if the target search area in the current frame is three times larger than the initial target’s size, the search area and its position is reset to the one in the first frame.

**Run-time.** The algorithm was implemented in MATLAB 2013b, and the experiments were conducted on a machine with an Intel i5-3320M processor at 2.6 GHz clock speed and 8 GB RAM. Tracking speed using this hardware set up was approximately 33 ms.

### 3.7. Tracking by decision fusion

Fusing the results from different methods or annotations has shown improvements for various applications (Sinha *et al* 2008) including classification (Kittler *et al* 1998), segmentation (Rohlfing *et al* 2003, Heckemann *et al* 2006), and tracking. Therefore we included an investigation of such a fusion approach. The tracking results of all six previously described methods were combined by computing for each frame *t* the median position of the tracked points *P*_*j*_(*t*) from the automatic methods. Using the median helps to reduce the influence of outlier results. Furthermore we determined the performance when fusing only the two methods which had the lowest mean tracking errors on the training data.

## 4. Evaluation

We compared the performance of the methods described in section 3 on the test data, consisting of 66 point-landmarks (vessel centers) in 26 2D sequences and 28 point-landmarks (vessel bifurcations) in 13 3D sequences, which the observers were confident to be able to reliably annotate. We evaluated the performance of the segmentation method in section 3.5 (PhR) on 11 manually segmented tumors in 9 2D sequences. In the following we describe the evaluation scheme used to validate and quantify the tracking accuracy.

### 4.1. Point-landmark tracking error

We randomly selected at least 10% of the images from each sequence and manually annotated the corresponding position of the initial point *P*_*j*_(0) in each of these selected images $I(\hat{t})$, denoted as ${{\bar{P}}_{j}}(\hat{t})$. The number of annotated frames per sequence is listed in table 1. For the annotated frame $I(\hat{t})$ and landmark *j*, we calculated the tracking error (TE) as the Euclidean distance between the estimated landmark position ${{P}_{j}}(\hat{t})$ and its annotation (ground truth) ${{\bar{P}}_{j}}(\hat{t})$:

14 \begin{eqnarray*}T{{E}_{j}}(\hat{t})=\,\parallel {{P}_{j}}(\hat{t})-{{\bar{P}}_{j}}(\hat{t})\parallel .\end{eqnarray*}

We summarized the results by the mean (MTE), standard deviation (STD) and 95th percentile of the single distribution including all $\text{T}{{\text{E}}_{j}}(\hat{t})$ belonging to a particular subgroup. These subgroups were the individual landmarks *j* (MTE_*j*_), the image groups (MTE_ETH_, MTE_MED1_ and MTE_MED2_ for 2D sequences, and MTE_EMC_, MTE_ICR_ and MTE_SMT_ for 3D sequences) and the landmark dimensionality (MTE_2D_ and MTE_3D_).

We also included the motion magnitude of the landmarks, defined as:

15 \begin{eqnarray*}{{M}_{j}}=\,\parallel {{P}_{j}}(0)-{{\bar{P}}_{j}}(\hat{t})\parallel .\end{eqnarray*}

We estimated the inter-observer variability of the results. For this two additional experts annotated a randomly selected subset of the images marked by the first observer amounting to a total of 3% of all images. We then defined as ground truth the mean position over the three annotations and computed the tracking error as described above.

Per tracking task, the median results from all employed methods were pair-wise tested for statistically significantly differences at the probability level *p* = 0.001 using the sign test. The sign test was used as it is a non-parametric test, which neither assumes a normal distribution nor a symmetric distribution.

### 4.2. Segmentation accuracy

A clinical expert segmented the visible boundaries of the tumors corresponding to the reference segmentation in each frame *t* of the 2D OX sequences. The results of the segmentation tracking were quantified by computing the Dice coefficient:

16 \begin{eqnarray*}\text{Dic}{{\text{e}}_{j}}\left({{\bar{S}}_{j}}(t),{{S}_{j}}(t)\right)=2\frac{\mid {{{\bar{S}}}_{j}}(t){}^{}{{S}_{j}}(t)\mid}{\mid {{{\bar{S}}}_{j}}(t)\mid \,+\,\mid {{S}_{j}}(t)\mid},\end{eqnarray*}

where ${{\bar{S}}_{j}}$ and *S*_*j*_ denote the manually segmented and the estimated tumor region, respectively, in each frame *t*. $\text{Dice}\in [0,100]$% measures the overlap ratio of the two regions and is at best 100%. We summarized the results by the mean, standard deviation and 5th percentile of the Dice coefficient distribution for each segmented tumor in the entire sequence. For comparison, we calculated the initial Dice coefficient, before tracking, as follows:

17 \begin{eqnarray*}\text{Dic}{{\text{e}}_{j}}\left({{\bar{S}}_{j}}(t),{{S}_{j}}(0)\right)=2\frac{\mid {{{\bar{S}}}_{j}}(t){}^{}{{S}_{j}}(0)\mid}{\mid {{{\bar{S}}}_{j}}(t)\mid \,+\,\mid {{S}_{j}}(0)\mid}.\end{eqnarray*}

## 5. Results

### 5.1. 2D landmarks

The results for the 2D point-landmark tracking are summarized in table 3. The MTE_2D_ ranges from 1.4 mm to 2.1 mm for the automatic methods, with best results achieved by MEVIS. Fusing the results of all tracking methods improved accuracy by 15–41% in comparison to the individual results, achieving a MTE_2D_ of 1.2 mm.

**Table 3.** Results of 2D point-landmark tracking.

Method	Tracking error			Mean error range of sequences		
	MTE_2D_	STD	95thTE	MTE_*j*∈ ETH_	MTE_*j*∈ MED1_	MTE_*j*∈ MED2_
Fusion	1.23	1.52	3.26	[0.36, 2.04]	[0.81, 7.83]	[0.89, 2.97]
MEVIS	1.44	2.04	3.86	[0.31, 2.61]	[0.90, 8.75]	[1.02, 3.47]
MEVIS + MED	1.53	2.45	3.95	[0.32, 3.02]	[0.94, 5.12]	[1.20, 12.71]
TUM	1.64	1.84	4.68	[0.43, 7.48]	[0.56, 4.24]	[0.95, 2.83]
KM	1.83	3.16	4.82	[0.37, 1.73]	[0.93, 13.22]	[1.62, 3.63]
PhR	2.00	2.87	5.59	[0.51, 3.47]	[0.79, 12.72]	[0.88, 3.54]
MEVIS + FOKUS	2.09	2.87	6.22	[0.52, 10.05]	[0.59, 11.27]	[0.88, 3.36]
Motion *M*	6.64	4.81	15.53	[2.90, 13.56]	[3.78, 12.48]	[4.33, 12.31]

*Note*: The results are in millimeters and ranked (top to bottom) according to increasing MTE_2D_.

Three of the proposed methods had for some sequences a higher mean error (MTE_*j*_) than the landmark motion *M*_*j*_.

To assess the robustness of all methods, we quantified the percentage of failures, i.e. the percentage of annotated frames per landmark for which $\text{TE}&gt;3$ mm or $\text{TE}&gt;5$ mm. These results, summarized in figure 6, show that the percentage of failures ranges between 6.3% and 15.8% (1.6% and 7.2%) for $\text{TE}&gt;3$ mm ($\text{TE}&gt;5$ mm), with the fusion method being the best one.

**Figure 4.**
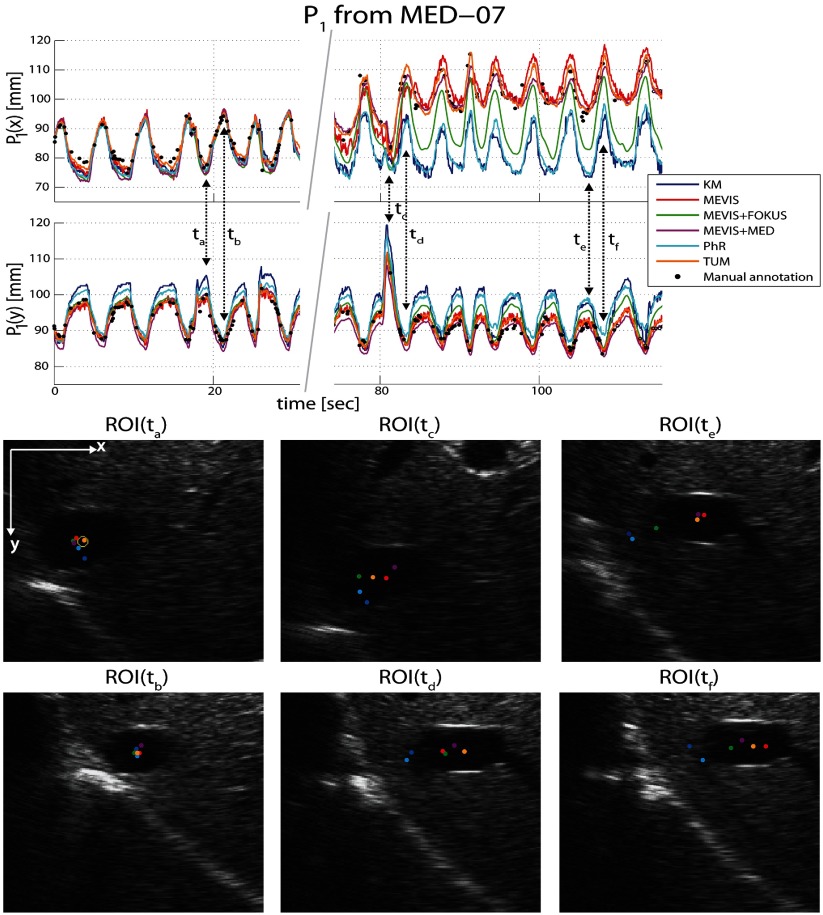
Illustration of tracking performance for landmark *P*_1_ from sequence MED-07. Tracking errors MTE_1∈*MED*−07_ were 13.22 (KM), 3.84 (MEVIS), 7.46 (MEVIS + FOKUS), 2.88 (MEVIS + MED), 12.72 (PhR) and 1.93 mm (TUM). The mean motion for the landmark was 11.23 mm. Frames at *t*_*a*_, *t*_*c*_ and *t*_*e*_ correspond to end-inhalations (with a deep inhale happening at *t*_*c*_), while *t*_*b*_, *t*_*d*_ and *t*_ *f* _ correspond to end-exhalations. In ROI(*t*_*a*_) the manual annotation is shown as a yellow circle.

We illustrate the difference in tracking performance between all methods for the point which had the highest variance of MTE (24.85 mm) across all methods, namely *P*_1_ from MED-07 (sequence details are listed in table 1). From figure 4 it can be observed that high tracking errors occur for some of the proposed methods when the landmark position drifted after a deep inhalation (at frame *t*_*c*_) of the subject under investigation. Comparing the associated ROIs, it seems that methods KM and PhR maintain a similar distance to the diaphragm, rather than stay with the vessel.

**Figure 5.**
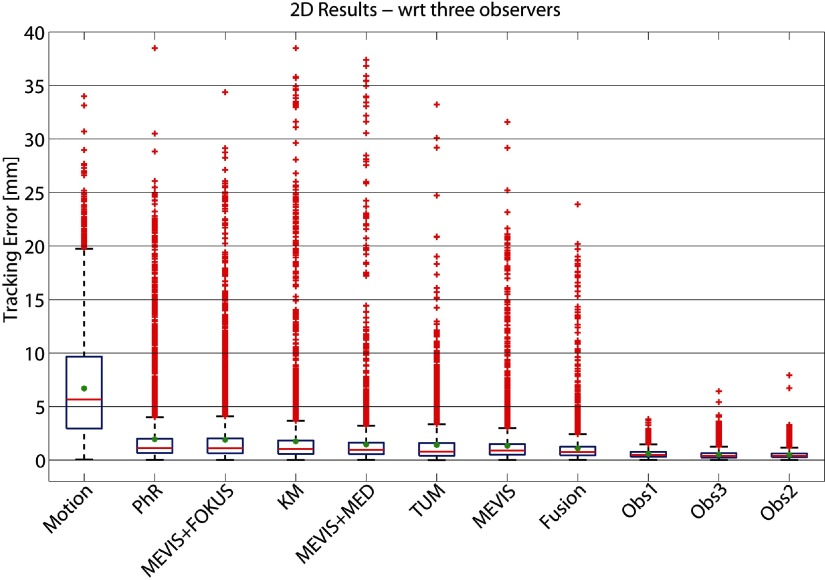
Box-plot summarizing the 2D tracking error (in mm) w.r.t. mean manual annotation of three observers (Obs). Results are ranked (left to right) according to decreasing MTE_2D_ (in green). On each box, the central red line is the median and the edges of the box are given by *q*1 = 25th and *q*3 = 75th percentiles of the error. Outliers are drawn as red crosses if larger than *q*3 + *w*(*q*3  −  *q*1), where *w* = 1.5 corresponds to approximately ±2.7 STD of the data.

Lower errors were obtained with respect to the mean annotation of the 3 observers with MTE_2D_ reduced by 0.06 to 0.23 mm for all methods, while the mean motion *M* was similar, see table 5. Note that now the order changed between MEVIS + MED and TUM and between PhR and MEVIS + FOKUS. The MTE_2D_ of the 3 observers was more than 50% lower than any individual method. An overview of these results is given in the box-plots in figure 5.

**Table 4.** Results of 3D point-landmark tracking.

Method	Tracking error			Mean error range of sequences		
	MTE_3D_	STD	95thTE	MTE_*j*∈ EMC_	MTE_*j*∈ ICR_	MTE_*j*∈ SMT_
Fusion	2.48	2.46	6.91	[1.19, 9.84]	[2.53, 2.59]	[0.94, 8.12]
PhR	2.55	2.46	7.98	[1.03, 9.63]	[2.54, 3.89]	[0.99, 11.57]
MEVIS + MED	2.71	3.01	7.58	[1.36, 10.40]	[1.59, 2.76]	[1.00, 6.59]
MEVIS + FOKUS	4.63	4.03	12.44	[2.41, 11.26]	[4.28, 5.88]	[1.23, 10.10]
Motion *M*	6.19	4.64	14.83	[3.59, 13.16]	[4.47, 5.72]	[2.46, 12.89]

*Note*: The results are in millimeters and ranked according to increasing MTE_3D_.

**Table 5.** Summary of the results of point-landmark tracking w.r.t. mean manual annotation of three observers.

Method	2D tracking error					3D tracking error			
	MTE_2D_	STD	MedianE	95thTE	MTE$_{2\text{D}}^{3\text{D}}$	MTE_3D_	STD	MedianE	95thTE
Fusion	1.08	1.42	0.75	2.85	1.32	2.43	2.76	1.49	7.61
KM	1.75	3.05	1.03	4.76	✗	✗	✗	✗	✗
MEVIS	1.33	1.94	0.88	3.56	✗	✗	✗	✗	✗
MEVIS + FOKUS	1.90	2.75	1.11	6.02	2.33	4.79	4.72	2.99	13.48
MEVIS + MED	1.45	2.48	0.95	3.49	1.78	2.76	4.10	1.52	8.80
PhR	1.94	2.93	1.12	5.53	2.38	2.83	2.97	1.46	9.67
TUM	1.41	1.89	0.78	4.70	✗	✗	✗	✗	✗
Motion *M*	6.69	4.78	5.65	15.52	8.19	5.83	4.21	4.52	14.80
Obs1	0.58	0.42	0.48	1.40	0.71	1.21	1.21	0.79	4.43
Obs2	0.48	0.34	0.41	1.06	0.59	1.73	2.66	0.78	8.74
Obs3	0.50	0.41	0.40	1.23	0.61	1.81	2.61	0.95	5.61

*Note*: The results are in millimeters and ranked according to alphabetical order of the methods.

**Figure 6.**
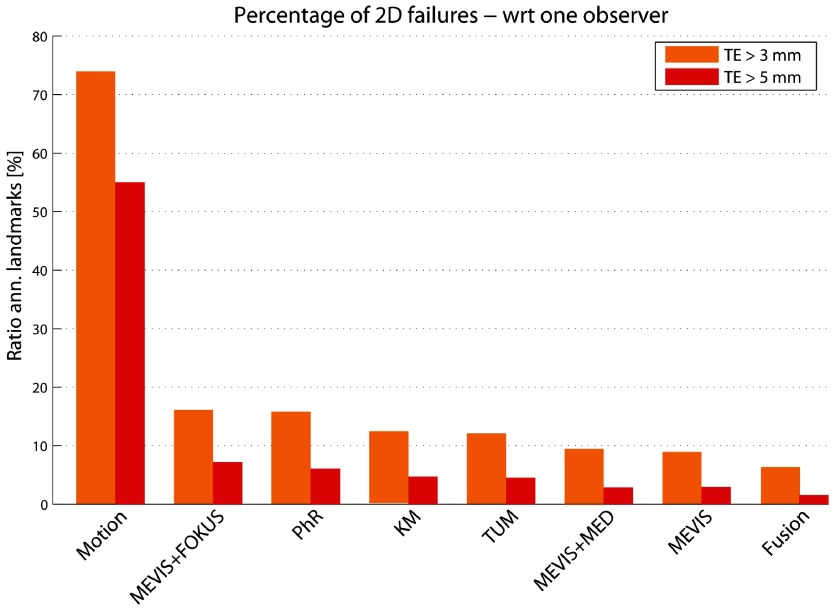
Percentage of failure cases: ratio of annotated 2D landmarks whose $\text{TE}&gt;3$ mm (orange) or $\text{TE}&gt;5$ mm (red) shown for all methods. Results are shown (left to right) according to decreasing MTE_2D_ (see table 3). TE is evaluated with respect to one observer.

There was low correlation between the motion magnitude of the landmarks and the tracking errors. In details, the sample Pearson correlation coefficients ($\rho $) between landmark motion and tracking errors for the individual methods ranged from 0.11 to 0.37, while for the observers there was no correlation ($\rho \in [0.06,0.11]$).

We also studied if tracking errors were influenced by the change in image quality due to the range of center frequencies used during the US acquisitions (see table 1). Only low correlation ($\rho &lt;0.43$) was found between MTE_*j*∈2D_ of the individual methods and center frequency of landmark *j*.

Tracking landmarks close to the acquisition border can be difficult. We analyzed the correlation between the landmark distance to the acquisition border and the tracking error for the landmark which comes closest to the acquisition border (*P*_2_ from MED-08), and found a high correlation ($\rho &gt;0.70$) for all methods except TUM ($\rho =0.12$).

The median results with respect to the mean annotation of the 3 observers from all methods were significantly different to each other, except for ${{p}_{\text{Fusion},\text{TUM}}}=0.003$ and ${{p}_{\text{MEVIS}+\text{FOKUS},\text{PhR}}}=0.655$. The median tracking errors of the observers were similar, apart from Obs1’s being significantly higher.

### 5.2. 3D landmarks

Results for all methods on the 3D sequences are shown in table 4. On average, the most accurate results were achieved by PhR, with MTE_3D_ of 2.55 mm. The fusion method slightly improved the average results by 3%. The percentages of failures are shown in figure 8 for all methods. These ranged between 18.7% and 51.3% (6.8% and 29.6%) for $\text{TE}&gt;3$ mm (5 mm).

**Figure 7.**
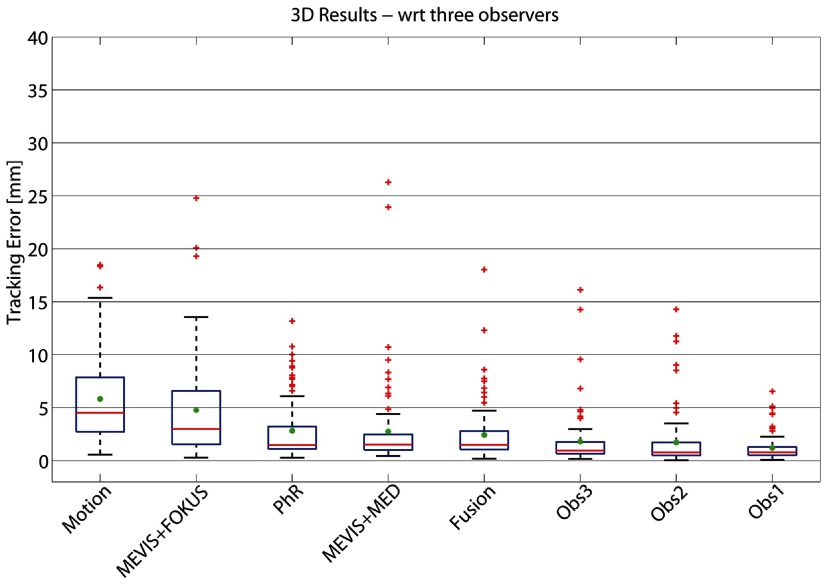
Box-plot summarizing the 3D tracking error (in mm) w.r.t. mean manual annotation of three observers (Obs). Results are ranked (left to right) according to decreasing MTE_3D_ (in green). On each box, the central red line is the median and the edges of the box are given by *q*1 = 25th and *q*3 = 75th percentiles of the error. Outliers are drawn as red crosses if larger than *q*3 + *w*(*q*3  −  *q*1), where *w* = 1.5 corresponds to approximately ±2.7 STD of the data.

The tracking errors with respect to the mean manual annotation of 3 observers are increased by 0.05–0.28 mm, while the mean motion is reduced by 0.36 mm. The error obtained by fusing all results is slightly reduced, see table 5. An overview of these results is given in the box-plots in figure 7.

**Figure 8.**
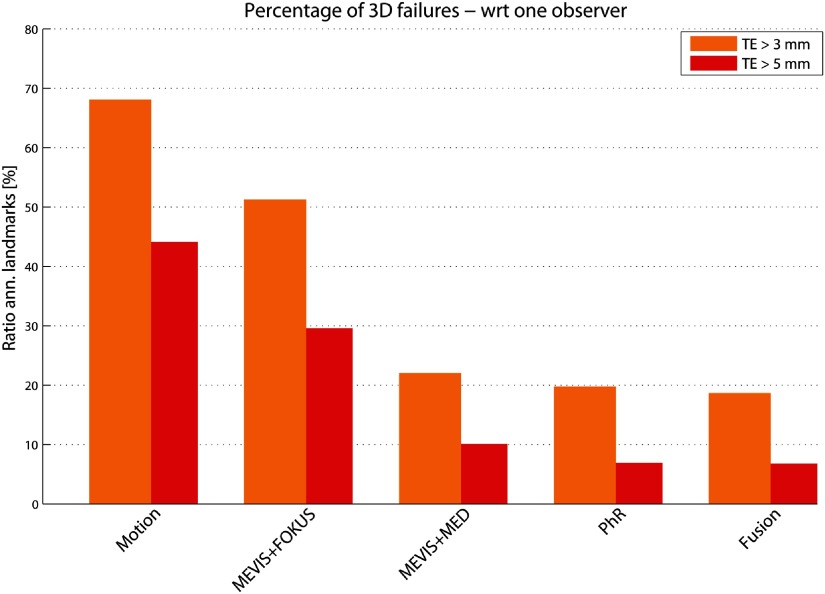
Percentage of failure cases: ratio of annotated 3D landmarks whose $\text{TE}&gt;3$ mm (orange) or $\text{TE}&gt;5$ mm (red) shown for all methods. Results are shown (left to right) according to decreasing MTE_3D_ (see table 4). TE is evaluated with respect to one observer.

**Figure 9.**
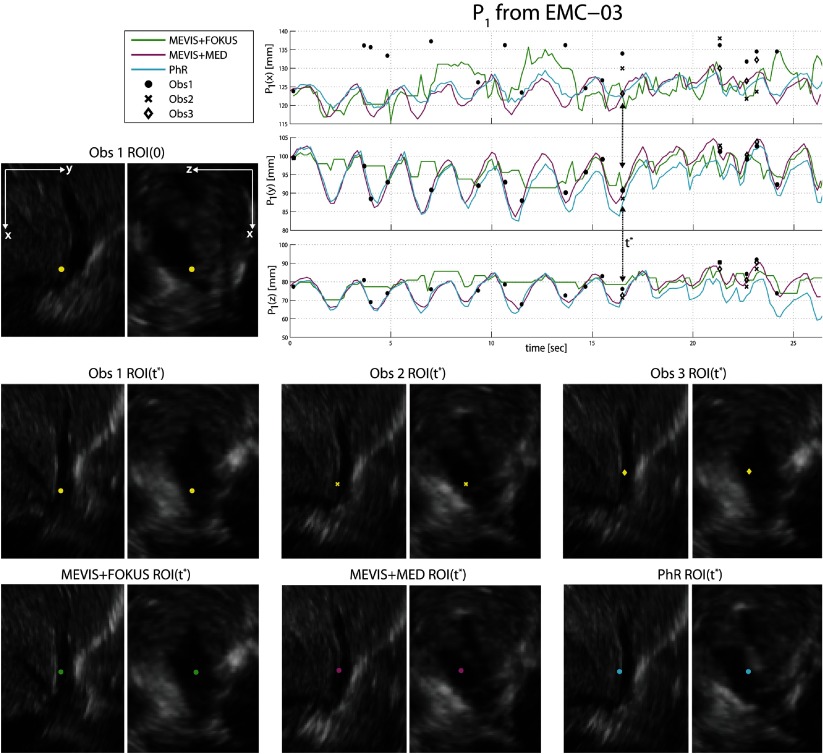
Illustration of tracking performance for landmark *P*_1_ from sequence EMC-03. Tracking errors MTE_1∈EMC−03_ with respect to the mean of 3 observers were 7.77 mm (MEVIS + FOKUS), 5.63 mm (MEVIS + MED) and 9.93 mm (PhR). The mean motion for the landmark was 11.71 mm. Inter-observer errors were 4.06 mm (Obs1), 11.59 mm (Obs2) and 9.76 mm (Obs3). The tracking results and annotations are shown at time ${{t}^{*}}$ for the same ROI(${{t}^{*}}$) with planes cut at the corresponding ${{P}_{1}}\left({{t}^{*}}\right)$ from each method.

Figure 9 shows the result with the highest MTE_*j*_ for MEVIS + MED and PhR. Tracking methods and observers disagreed mainly along the vessel (x-coordinate), showing the challenge of tracking landmarks within elongated structures without a stable bifurcation.

All MTE_3D_ were larger than the estimated equivalent 3D error from MTE_2D_^16^16The mean 3D error was estimated from MTE_2D_ by $\text{MTE}_{2\text{D}}^{3\text{D}}=\sqrt{3\left(\text{MTE}_{2\text{D}}^{2}/2\right)}$, which assumes equal error components. (e.g. +70% Obs1, 84% Fusion, 55% MEVIS + MED), while the estimated 3D motion was on average lower (−29%). Yet this clear relationship was not sustained for error measures calculated in pixels (see table 6). In particular Fusion resulted in very similar results.

**Table 6.** Summary of the results of point-landmark tracking w.r.t. mean manual annotation of three observers.

Method	2D tracking error					3D tracking error			
	MTE_2D_	STD	MedianE	95thTE	MTE$_{2\text{D}}^{3\text{D}}$	MTE_3D_	STD	MedianE	95thTE
Fusion	2.68	3.52	1.81	7.02	3.28	3.35	3.77	2.14	10.02
KM	4.33	7.54	2.41	11.86	✗	✗	✗	✗	✗
MEVIS	3.29	4.73	2.10	8.89	✗	✗	✗	✗	✗
MEVIS + FOKUS	4.72	7.00	2.75	15.85	5.78	6.34	6.13	3.94	16.78
MEVIS + MED	3.68	6.89	2.32	8.86	4.51	3.66	5.12	2.11	11.36
PhR	4.71	7.12	2.71	13.56	5.77	3.80	3.88	2.09	13.22
TUM	3.37	4.39	1.98	11.48	✗	✗	✗	✗	✗
Motion *M*	16.91	12.49	14.13	40.39	20.71	8.00	5.82	6.03	21.00
Obs1	1.45	1.09	1.12	3.66	1.78	1.71	1.56	1.14	4.56
Obs2	1.19	0.87	0.99	2.70	1.46	1.60	1.55	1.11	5.11
Obs3	1.25	1.05	0.98	3.11	1.53	1.82	1.56	1.36	4.69

*Note*: The results are in pixels/voxels and ranked according to alphabetical order of the methods.

The median error (see table 5) of MEVIS + FOKUS was statistically different (*p*  <  0.001) with respect to the one of Fusion and PhR. No other significant difference between the methods existed. The median tracking errors of the 3 observers were statistically significantly lower than the ones from the 4 tracking methods, but not significantly different amongst each other.

We investigated if the landmark error is correlated with the motion and found little evidence for such a correlation, as the sample Pearson correlation coefficients $\rho $ was in the range of 0.25–0.45 for all methods and observers, apart from MEVIS + FOKUS ($\rho =0.62$).

For 3D, a low to moderate correlation ($\rho \in [0.26,0.51]$) was found between tracking errors (MTE_*j*∈3*D*_) and center frequency of the US acquisition protocol (see table 1) for each landmark *j*.

We analyzed the correlation between the mean tracking error and the distance to the acquisition border for a 3D vessel bifurcation landmark, which was moving close to this border and had good local image contrast throughout the sequence. We found that the distance was moderately correlated with the TE of landmark *P*_1_ from SMT-02 for all methods ($\rho \in [0.47,0.58]$).

### 5.3. 2D segmentations

The results of the tumor segmentation task, performed by PhR are summarized in table 7. The segmentation accuracy, expressed in mean Dice coefficient, ranges between 76.7% and 92.3%. The mean accuracy is 8% higher than the initial overlap ratio, but lower in 3 sequences.

**Table 7.** Results of 2D tumor segmentation tracking (PhR) w.r.t. manual annotation of a clinical expert.

Segmentation	Tracking overlap [%]			Initial Dice [%]		
	Mean	STD	5th TO	Mean	STD	5th TO
OX-1, *S*_1_	86.6	5.4	78.4	49.9	25.3	20.6
OX-2, *S*_1_	85.5	4.8	76.6	73.7	10.7	57.1
OX-4, *S*_1_	92.3	1.9	90.0	74.9	17.6	46.3
OX-5, *S*_1_	79.8	6.6	68.4	87.3	5.5	77.3
OX-6, *S*_1_	76.7	9.2	58.6	72.6	12.7	51.9
OX-7, *S*_1_	89.6	4.3	78.8	80.5	6.2	71.5
OX-7, *S*_2_	77.2	4.8	70.0	58.3	15.5	33.2
OX-8, *S*_1_	88.7	2.7	84.9	92.1	3.5	84.9
OX-9, *S*_1_	92.2	2.2	88.8	91.8	5.0	81.9
OX-9, *S*_2_	77.6	5.7	66.7	80.2	8.2	65.7
OX-10, *S*_1_	81.8	3.4	76.7	75.5	6.8	63.2
OX	84.1	7.5	71.1	77.8	16.7	41.5

*Note*: The results are in % of the Dice coefficient.

**Figure 10.**
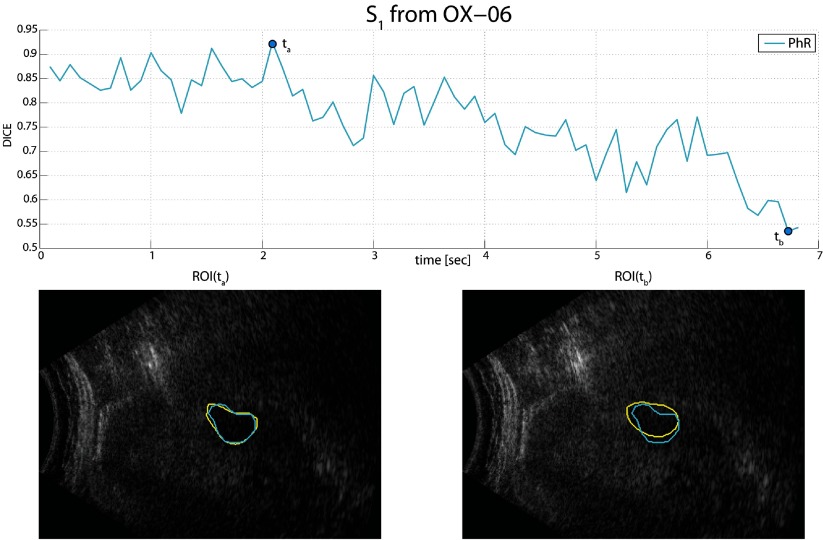
Illustration of tracking performance for *S*_1_ from OX-6. The Dice coefficient ranged from 92.5 % (at *t*_*a*_) to 53.6% (at *t*_*b*_). ROI(*t*_*a*_) and ROI(*t*_*b*_) show the overlap of the manual (in yellow) and PhR (in light blue) segmentations.

Figure 10 shows the results of the segmentation on the sequence with the highest variance of the Dice coefficient, i.e. *S*_1_ from OX-6. The overlap gradually decreases, likely due to lower image contrast compared to the other sequences.

## 6. Discussion

When considering the individual methods (described in sections 3.1–3.6), determining the local translations of the vessels by a Bayesian approach (MEVIS + MED) was computationally the most efficient algorithm and was runner-up (by 6%) in both landmark tasks. The Sparse Demons method on patches (PhR) performed best for the 3D landmarks, but had a relative poor performance for the 2D landmarks. For the case illustrated in figure 5, the method tracks the diaphragm as this is the main bright feature in the reference patch, resulting in high errors when the distance to the vessel changes. The variational registration approach within relative large regions (MEVIS) provided on average the most accurate results for the 2D landmarks, but was unfortunately not tested for the 3D landmarks. Registration to the initial frame and if needed to the previous frame, allowing smooth deformations, as well as including NGF in the image similarity likely contributed to this good performance.

Fusion of the tracking results from all the independent methods by taking their median value provided on average the highest landmark tracking accuracy, with improvements by at least 15% (3%) for 2D (3D) landmarks. This reduced mean motion by 82% (60%) resulting in a mean accuracy of 1.2 mm (2.5 mm). Yet it requires the run-time of the slowest method and *n* times the computing power, where *n* is the number of fused methods (in this paper *n* = 6 for 2D and *n* = 3 for 3D). To save resources, fusing only the 2 methods, which performed on average best on the training set, led to worse results for 2D (MTE_2D_=1.6 mm for median of MEVIS + MED and MEVIS + FOKUS) as the training performance of MEVIS + FOKUS was not representative. More training data will hopefully enable selecting instead one of the better methods (e.g. MEVIS), which would have led to a slightly better fusion result (1.4 mm). Alternatively fusing more methods might circumvent this problem (e.g. incorporating MEVIS as third best from training data would result in an MTE of 1.3 mm). For 3D, similar results were achieved, with the median of MEVIS + MED and PhR lead to MTE_3D_=2.5 mm.

The tracking performance was generally not dependent on the motion magnitude. Comparing the errors in millimeters, tracking the 3D landmarks appears to be a harder task than tracking the 2D landmarks. This can mostly be explained by the lower image resolution, since 2D and 3D results from Fusion are very similar when considering the pixel resolution and assuming equal errors in each dimension (2D: 2.7 pixels, estimated 3D: 3.3 pixels, 3D: 3.4 pixels). Hence it seems very likely that advances in 3D image resolution will improve tracking results. Other factors include some worse image quality (e.g. EMC), a more cumbersome annotation task and greater difficulties of visually inspecting the 3D images for parameter optimization.

Methods that adjusted their parameters per sequence for the MICCAI 2014 challenge were TUM, MEVIS, MEVIS + MED and MEVIS + FOKUS (only 2D case). The mean performance of these ‘tuned’ methods for all 2D test data was worse (TUM 1.84 mm, MEVIS 1.51 mm), similar (MEVIS + MED 1.52 mm), or better (MEVIS + FOKUS 1.91 mm) than with fixed parameters (table 3). For the 3D case, resubmission was needed only from MEVIS + MED, for which the original submission resulted in worse accuracy (MTE_3D_=2.80 mm) than fixed parameters (table 4).

We compared the methods to previously published results (Cifor *et al* 2013, De Luca *et al* 2013, Vijayan *et al* 2014) by re-computing the tracking error for the same subset (excluding any training data), the same error measure and annotations. For 2D landmark tracking, two of the individual methods performed on average better than previously published results, namely MEVIS + MED achieved an MTE (95th TE) of 0.6 (1.4) mm and MEVIS of 0.7 (1.6) mm, versus 0.8 (1.7) mm from De Luca *et al* (2013). This comparison was based on ETH-01 to ETH-09 without ETH-05. For 3D landmark tracking, comparison was done on the SMT subset. The method proposed in Vijayan *et al* (2014) achieved an MTE (95th TE) of 3.6 (14.9) mm. PhR and MEVIS + MED obtained on average better results, with an MTE (95th TE) of 2.4 (6.8) mm and 2.6 (7.3) mm respectively. The single method attempting the tracking of tumor regions performed similar to the previous baseline method (Cifor *et al* 2013). The comparison was possible only for 3 sequences, where PhR was on average 5% (OX-01) and 1% (OX-04) worse, but 4% better (OX-02) than the method from Cifor *et al* (2013).

On average, the tracking performance was worse than the observer annotation accuracy, which hints at potentials for further method improvements. Error reduction beyond the observer accuracy will require improvements in image resolution and quality. The clinically acceptable tracking error depends on the application. The American Association of Physicists in Medicine recommends for external-beam radiation therapy that respiratory management techniques are warranted when the target motion is greater than 5 mm (Keall *et al* 2006). This implies that techniques for predicting the target motion should have a maximum error of at most 5 mm. Tracking will need to be more accurate as errors are also introduced by temporal prediction to compensate system latencies and spatial prediction if the target cannot be directly tracked. Overall this was clearly not yet achieved by any automatic tracking methods (see figures 4, 6, 7 and 8) and also observers had some difficulties. Only for one method (MEVIS + MED) and the ICR sequences the tracking errors stayed always within the 5 mm limit (maximum TE: 4.7 mm), likely due to good image quality, smaller field of view, no disappearing structures, and high temporal and spatial resolution.

The high 95th percentile errors and number of tracking failures indicate that the methods are not very robust. Visual inspection of failure cases hint at problems due to accumulation of errors, disappearing structures at the image acquisition border, out-of-plane motion, substantial changes in image appearance or insufficient motion capture ranges. These might be the reasons of the high variance in the individual tracking performances across the sequences.

Tracking run-times per frame range from 1.8 ms to 84 ms for 2D and from 51 ms to about 100 ms for 3D. Processing times generally varied depending on the number of landmarks and the image appearance. Real-time performance was achieved by 4 methods for 2D and 3 for 3D landmarks (MEVIS + MED, MEVIS + FOKUS, PhR and TUM), assuming average acquisition times of 50 ms (20 Hz) and 125 ms (8 Hz). The remaining methods are not far off, indicating that further code optimization and hardware improvements are likely to provide real-time performance for the current dataset. Furthermore, off-line applications, such as dose accumulation during radiation therapy, do not require real-time performance. Hence, the fusion of several tracking results can be a promising approach, despite having the cumulative computational burden of all fused methods (which is however easily parallelizable), as it achieved the highest accuracy.

For the purpose of evaluating different tracking approaches, specific anatomical landmarks in the first frame were provided in CLUST. These landmarks were manually selected after inspecting the US images, to ensure that they would not disappear during free-breathing. In clinics, real-time applications should require as little manual interventions as possible. Therefore, tracking algorithms might benefit from incorporating the automatic detection of stable features to track throughout the treatment and update tracking regions when necessary. Feature detection might be based on analyzing the tracking results from an initial set of images covering a few breathing cycles. Testing this functionality is outside the CLUST challenge, as the aim is to compare tracking performances for the same landmarks.

## 7. Conclusion

This paper describes the results of the MICCAI 2014 challenge on liver US tracking (CLUST14), which enabled for the first time the quantitative, direct comparison of tracking methods for this application.

The challenge data included a large number of realistic sequences, which varied in length, spatial and temporal resolution, acquisition settings and US scanner.

Quantitative evaluation of all results showed a mean tracking error from 1.4 mm to 2.1 mm for 2D points, and from 2.6 mm to 4.6 mm for 3D points. Considering the median tracking results of all methods improved the mean error to 1.2 mm (2D) and 2.5 mm (3D). The segmentation task, fulfilled only by one participant, resulted in a mean Dice coefficient of 84.1%. All best approaches are comparable or better than the state-of-the-art.

Applicability for therapy guidance still requires general improvement of the 3D landmark tracking accuracy as well as reduction of tracking failures ($\text{TE}&gt;5$ mm). Advances in image resolution and quality will support this task. Furthermore the diaphragm is a prominent feature in liver US images which should also be tracked.

For some sequences, the variability of the observers was particularly high, due to the difficulty in manually annotating 3D volumes. Having more observers might lead to a more reliable reference for computing the tracking results.

Most of the methods achieved, or are close to, real-time, while running on standard machines.

In conclusion, this CLUST14 challenge provided a good basis for a first comparison of US tracking methods for the liver. Its accompanying workshop facilitated lively discussions of the involved researchers. The research community can benefit from this benchmark and the CLUST challenge remains open for future participants to evaluate their method and be included in the online leader board. Future work includes the increase of the number of sequences and tracking of other available structures in the liver, e.g. the diaphragm.

## References

[pmb515767bib001] Arulampalam M S, Maskell S, Gordon N, Clapp T (2002). A tutorial on particle filters for online nonlinear/non-Gaussian Bayesian tracking. IEEE Trans. Signal Process..

[pmb515767bib002] Banerjee J, Klink C, Peters E D, Niessen W J, Moelker A, van Walsum T (2015). Fast and robust 3D ultrasound registration—block and game theoretic matching. Med. Image Anal..

[pmb515767bib003] Banerjee J, Klink C, Peters E D, Niessen W J, Moelker A, van Walsum T (2014). 4D liver ultrasound registration. Lecture Notes in Computer Science.

[pmb515767bib004] Cifor A, Risser L, Chung D, Anderson E, Schnabel J (2012). Hybrid feature-based Log-Demons registration for tumour tracking in 2D liver ultrasound images.

[pmb515767bib005] Cifor A, Risser L, Chung D, Anderson E, Schnabel J (2013). Hybrid feature-based diffeomorphic registration for tumor tracking in 2D liver ultrasound images. IEEE Trans. Med. Imaging.

[pmb515767bib006] Comaniciu D, Ramesh V, Meer P (2003). Kernel-based object tracking. IEEE Trans. Pattern Anal. Mach. Intell..

[pmb515767bib007] De Luca V, Tanner C, Szekely G (2012). Speeding-up image registration for repetitive motion scenarios.

[pmb515767bib008] De Luca V, Tschannen M, Szekely G, Tanner C (2013). A learning-based approach for fast and robust vessel tracking in long ultrasound sequences. Medical Image Computing and Computer-Assisted Intervention.

[pmb515767bib009] Epanechnikov V A (1969). Non-parametric estimation of a multivariate probability density. Theory Probab. Appl..

[pmb515767bib010] Farnebäck G (2003). Two-frame motion estimation based on polynomial expansion. Image Analysis.

[pmb515767bib011] Fischer B, Modersitzki J (2003). Curvature based image registration. J. Math. Imaging Vision.

[pmb515767bib012] Fukunaga K, Hostetler L (1975). The estimation of the gradient of a density function, with applications in pattern recognition. IEEE Trans. Inf. Theory.

[pmb515767bib013] Haber E, Modersitzki J (2006). Intensity gradient based registration and fusion of multi-modal images. Medical Image Computing and Computer-Assisted Intervention (Lecture Notes in Computer Science).

[pmb515767bib014] Harris E, Miller N, Bamber J C, Symonds-Tayler J, Evans P (2010). Speckle tracking in a phantom and feature-based tracking in liver in the presence of respiratory motion using 4D ultrasound. Phys. Med. Biol..

[pmb515767bib015] Heckemann R A, Hajnal J V, Aljabar P, Rueckert D, Hammers A (2006). Automatic anatomical brain MRI segmentation combining label propagation and decision fusion. NeuroImage.

[pmb515767bib016] Isard M, Blake A (1996). Contour tracking by stochastic propagation of conditional density. Computer Vison.

[pmb515767bib017] Keall P (2006). The management of respiratory motion in radiation oncology report of AAPM task group 76. Med. Phys..

[pmb515767bib018] Kittler J, Hatef M, Duin R, Matas J (1998). On combining classifiers. IEEE Trans. Pattern Anal. Mach. Intell..

[pmb515767bib019] König L, Kipshagen T, Rühaak J (2014). A non-linear image registration scheme for real-time liver ultrasound tracking using normalized gradient fields.

[pmb515767bib020] König L, Rühaak J (2014). A fast and accurate parallel algorithm for non-linear image registration using normalized gradient fields.

[pmb515767bib021] Lediju Bell M A, Byram B C, Harris E J, Evans P M, Bamber J C (2012). *In vivo* liver tracking with a high volume rate 4D ultrasound scanner and a 2D matrix array probe. Phys. Med. Biol..

[pmb515767bib022] Lediju M A, Byram B C, Harris E J, Evans P M, Bamber J C (2010). 3D liver tracking using a matrix array: implications for ultrasonic guidance of IMRT.

[pmb515767bib023] Lübke D, Grozea C (2014). High performance online motion tracking in abdominal ultrasound imaging.

[pmb515767bib024] Mansi T, Pennec X, Sermesant M, Delingette H, Ayache N (2011). iLogDemons: a demons-based registration algorithm for tracking incompressible elastic biological tissues. Int. J. Comput. Vision.

[pmb515767bib025] Matthews I, Ishikawa T, Baker S (2004). The template update problem. IEEE Trans. Pattern Anal. Mach. Intell..

[pmb515767bib026] McClelland J R, Hawkes D J, Schaeffter T, King A P (2013). Respiratory motion models: a review. Med. Image Anal..

[pmb515767bib027] Modersitzki J (2009). FAIR: Flexible Algorithms for Image Registration.

[pmb515767bib028] Mory B, Somphone O, Prevost R, Ardon R (2012). Real-time 3D image segmentation by user-constrained template deformation. Medical Image Computing and Computer-Assisted Intervention (Lecture Notes in Computer Science).

[pmb515767bib029] Ning J, Zhang L, Zhang D, Wu C (2012). Scale and orientation adaptive mean shift tracking. Comput. Vision.

[pmb515767bib030] Preiswerk F, De Luca V, Arnold P, Celicanin Z, Petrusca L, Tanner C, Bieri O, Salomir R, Cattin P C (2014). Model-guided respiratory organ motion prediction of the liver from 2D ultrasound. Med. Image Anal..

[pmb515767bib031] Rohlfing T, Russakoff D B, Maurer C R (2003). Expectation maximization strategies for multi-atlas multi-label segmentation. Information Processing in Medical Imaging (Lecture Notes in Computer Science).

[pmb515767bib032] Shirato H, Shimizu S, Kitamura K, Onimaru R (2007). Organ motion in image-guided radiotherapy: lessons from real-time tumor-tracking radiotherapy. Int. J. Clin. Oncol..

[pmb515767bib033] Sinha A, Chen H, Danu D, Kirubarajan T, Farooq M (2008). Estimation and decision fusion: a survey. Neurocomputing.

[pmb515767bib034] Somphone O (2013). Fast myocardial motion and strain estimation in 3D cardiac ultrasound with Sparse Demons.

[pmb515767bib035] Tanner C, Boye D, Samei G, Szekely G (2012). Review on 4D models for organ motion compensation. Crit. Rev. Biomed. Eng..

[pmb515767bib036] Thirion J P (1998). Image matching as a diffusion process: an analogy with Maxwell’s demons. Med. Image Anal..

[pmb515767bib037] Vijayan S, Klein S, Hofstad E F, Lindseth F, Ystgaard B, Langø T (2014). Motion tracking in the liver: validation of a method based on 4D ultrasound using a nonrigid registration technique. Med. Phys..

[pmb515767bib038] Vijayan S, Klein S, Hofstad E, Lindseth F, Ystgaard B, Langø T (2013). Validation of a non-rigid registration method for motion compensation in 4D ultrasound of the liver.

[pmb515767bib039] Zhang X, Günther M, Bongers A (2010). Real-time organ tracking in ultrasound imaging using active contours and conditional density propagation. Medical Imaging and Augmented Reality (Lecture Notes in Computer Science).

